# Anxiolytic-like effects of hochuekkito in lipopolysaccharide-treated mice involve interleukin-6 inhibition

**DOI:** 10.3389/fphar.2022.890048

**Published:** 2022-08-12

**Authors:** Soichiro Ushio, Yudai Wada, Mizuki Nakamura, Daiki Matsumoto, Kota Hoshika, Shoya Shiromizu, Naohiro Iwata, Satoru Esumi, Makoto Kajizono, Yoshihisa Kitamura, Toshiaki Sendo

**Affiliations:** ^1^ Department of Pharmacy, Okayama University Hospital, Okayama, Japan; ^2^ Department of Clinical Pharmacy, Okayama University Graduate School of Medicine, Dentistry and Pharmaceutical Sciences, Okayama, Japan; ^3^ Department of Pharmacotherapy, School of Pharmacy, Shujitsu University, Okayama, Japan

**Keywords:** anxiolytic, inflammation, immunomodulation, macrophages, Kampo medicine

## Abstract

Hochuekkito (HET) is a Kampo medicine used to treat postoperative and post-illness general malaise and decreased motivation. HET is known to regulate immunity and modulate inflammation. However, the precise mechanism and effects of HET on inflammation-induced central nervous system disorders remain unclear. This study aimed to assess the effect of HET on inflammation-induced anxiety-like behavior and the mechanism underlying anxiety-like behavior induced by lipopolysaccharide (LPS). Institute of Cancer Research mice were treated with LPS (300 μg/kg, intraperitoneally), a bacterial endotoxin, to induce systemic inflammation. The mice were administered HET (1.0 g/kg, orally) once a day for 2 weeks before LPS treatment. The light-dark box test and the hole-board test were performed 24 h after the LPS injection to evaluate the effects of HET on anxiety-like behaviors. Serum samples were obtained at 2, 5, and 24 h after LPS injection, and interleukin-6 (IL-6) levels in serum were measured. Human and mouse macrophage cells (THP-1 and RAW264.7 cells, respectively) were used to investigate the effect of HET on LPS-induced IL-6 secretion. The repeated administration of HET prevented anxiety-like behavior and decreased serum IL-6 levels in LPS-treated mice. HET significantly suppressed LPS-induced IL-6 secretion in RAW264.7 and THP-1 cells. Similarly, glycyrrhizin, one of the chemical constituents of HET, suppressed LPS-induced anxiety-like behaviors. Our study revealed that HET ameliorated LPS-induced anxiety-like behavior and inhibited IL-6 release *in vivo* and *in vitro*. Therefore, we postulate that HET may be useful against inflammation-induced anxiety-like behavior.

## Introduction

Inflammation is the body’s initial response to infection and tissue injury and has been shown to play an important role in the development of neurodegenerative diseases and psychiatric disorders, such as anxiety, bipolar disorder, schizophrenia, and depression (Berk et al., 2011; Leonard and Maes 2012; Yan et al., 2014; Khandaker et al., 2015; Månsson et al., 2022). The serum levels of pro-inflammatory cytokines, including interleukin-6 (IL-6), were higher in depressed patients with schizophrenia, bipolar disorder, and major depressive disorder (Dowlati et al., 2010; Goldsmith et al., 2016). In animal models, inflammatory conditions caused by the peripheral administration of lipopolysaccharide (LPS), which causes systemic inflammation through increased production of interferon-gamma (IFN-γ), IL-6, and interleukin-1 beta (IL-1β), have been shown to lead to both depressive-like and anxiety-like behaviors (Godbout et al., 2005; Huang et al., 2008; Jangra et al., 2014). Our previous study revealed that the systemic administration of LPS increased serum IL-6 levels and hippocampal mRNA levels of IL-6 and tumor necrosis factor-alpha (TNF-α) (Kitamura et al., 2019). Furthermore, the reduction of blood cytokine levels has been suggested as a novel therapy for inflammation-induced psychotic disorders (Zhang et al., 2017).

The light-dark test and the hole-board test is an anxiogenic challenge for measuring the response of an animal model to an unfamiliar environment and has been used to examine the effects of benzodiazepine anxiolytics, such as diazepam, on their emotional behavior (Takeda et al., 1998; Bourin and Hascoët 2003). The typical benzodiazepine receptor agonist diazepam is a widely prescribed anxiolytic drug. Using the hole-board test, we had previously reported that diazepam exhibited anxiolytic-like effects (Matsumoto et al., 2021). Therefore, diazepam showed improvement in anxiety-like behavior in the LPS-treated mice.

It was reported that 92.7% of Japanese family physicians have prescribed Kampo medicine (Takayama et al., 2020). Kampo medicine can treat a wide variety of conditions from mental disorders to physical weakness. Kampo medicine, including crude drugs of saiko such as hochuekkito (HET, Bu Zhong Yi Qi Tang in Chinese, Bo jung ik gi tang in Korea), shigyakusan, shosaikoto, and yokukansan (YKS, Yi Gan San in Chinese, Ukgansan in Korea), has been frequently prescribed for the treatment of a wide variety of conditions from mental disorders to physical weakness. Especially, HET was developed considering its anti-stress effects related to an extreme situation in ancient China, but it continues to be useful for dealing with the stress of fear and its subsequent depressive condition (Koshikawa et al., 1998). In recent years, it has become clear that chronic environmental stress induces neural inflammation together with depression and anxiety (Nie et al., 2018). Previous studies have reported that HET prevents systemic inflammation in patients with chronic obstructive pulmonary disease (Tatsumi et al., 2009). Furthermore, HET has been shown to attenuate LPS-induced inflammation in a mouse model of lung emphysema (Tatsumi et al., 2009; Isago et al., 2021). It improves cachexia by inhibiting cytokine secretion in cancer-bearing mice (Yae et al., 2012). Therefore, we speculated that this drug may improve inflammatory reactions and inflammation-induced psychotic disorders. The effect of HET use in psychiatric disorders has not been investigated previously. In this study, we aimed to investigate the anxiolytic effect of HET on anxiety-like behavior in LPS-treated mice using the light-dark test and hole-board test. In addition, we assessed the effect of HET on IL-6 expression in LPS-treated mice and LPS-exposed human and mouse cell lines.

## Materials and methods

### Animals

This study was conducted in accordance with the recommendations of the Guide for Animal Experiments of the Advanced Science Research Center, Okayama University. The animal study protocol was approved by the Animal Care Use Committee of the Advanced Science Research Center, Okayama University (OKU-2020565). A total of 234 male Institute of Cancer Research (ICR) mice weighing 29–35 g were purchased from Charles River Laboratories (Yokohama, Japan). The mice were housed in an air-conditioned room (23 ± 1°C with approximately 60% humidity) in groups of five per cage under a constant light-dark cycle (lights on from 08:00–20:00) and fed standard laboratory food and tap water.

### Drugs

LPS (from *Escherichia coli* O127:B8; Sigma-Aldrich, St. Louis, MO, United States) was dissolved in saline solution and administered by intraperitoneal (ip) injection at a dose of 1 ml/kg. Control mice were administered the vehicle. HET (Lot No. 20160041010) was provided by Tsumura & Co. (Tokyo, Japan). HET was used in the form of an extract powder made of the following raw materials: 4.0 parts Japanese Pharmacopoeia (JP) *Astragalus* root [*Astragalus membranaceus* Bunge, or *Astragalus mongholicus* Bunge (*Leguminosae*), radix], 4.0 parts JP Atractylodes lancea rhizome [*Atractylodes lancea* De Candolle, or *Atractylodes schinensis* Koidzumi (Asteraceae), hizome], 4.0 parts JP Ginseng [*Panax ginseng* C. A. Meyer (*Panax schinseng Nees*) (*Araliaceae*), radix], 3.0 parts JP Japanese angelica root [*Angelica acutiloba* Kitagawa, or *Angelica acutiloba* Kitagawa var. *sugiyamae* Hikino (*Umbelliferae*), radix], 2.0 parts JP Bupleurum root [*Bupleurum falcatum* Linné (*Umbelliferae*), radix], 2.0 parts JP Jujube [*Ziziphus jujuba* Miller var. *inermis* Rehder (*Rhamnaceae*), fructus], 2.0 parts JP Citrus unshiu peel [*Citrus unshiu* Marcowicz, or *Citrus reticulata* Blanco (*Rutaceae*), pericarpium], 1.5 parts JP *Glycyrrhiza* [*Glycyrrhiza uralensis* Fischer, or *Glycyrrhiza glabra* Linné (*Leguminosae*), radix], 1.0 parts JP Cimicifuga rhizome [*Cimicifuga simplex* Turczaninow, *Cimicifuga dahurica* Maximowicz, *Cimicifuga foetida* Linné, or *Cimicifuga heracleifolia* Komarov (*Ranunculaceae*), hizome], and 0.5 parts JP ginger [*Zingiber officinale* Roscoe (*Zingiberaceae*), hizome]. The plants were identified by their external morphology and marker compounds as per the Japanese pharmacopeia and company standards. The extract quality was standardized based on good manufacturing practices, as defined by the Ministry of Health, Labour, and Welfare of Japan. The 10 herbs were boiled in purified water at 95°C for 1 h. The liquid extract solution was filtered from the non-soluble waste and reduced to a concentrate. The concentrate was spray-dried to produce the extract powder. A three-dimensional high-performance liquid chromatogram of HET provided by Tsumura & Co. is presented in Supplementary Figure S1. The HET powder was dissolved in distilled water and doses were set based on a previous report (Tohda and Mingmalairak 2013; Cai and Yang 2019; Kitamura et al., 2019). Glycyrrhizin (Tokyo Chemical Industry CO., LTD, Tokyo, Japan) was suspended in water *in vivo* and doses were set based on a previous report (Song et al., 2013).

### *In vivo* experimental schedule

We assessed the preventive effect of the repeated administration of HET and glycyrrhizin on the LPS-induced anxiety-like behaviors in mice using the hole-board test and light-dark test. HET (0.1–1.0 g/kg) and glycyrrhizin (10–30 mg/kg) was administered orally (po) once a day for 2 weeks until the day before the experiments. Diazepam (0.1–1.0 mg/kg) was administered intraperitoneally (ip) 30 min before the experiments. The animals were then intraperitoneally injected with LPS (300 µg/kg) 1 h after the final HET and glycyrrhizin administration. The hole-board and light-dark box tests were performed 24 h after the LPS injection. Serum samples from mice were obtained 2, 5, and 24 h after the injection. The experiments were conducted between 10:00 and 16:00.

### Hole-board test

The hole-board test apparatus consisted of a gray box (50 × 50 × 50 cm) with 16 (4 × 4) equidistant holes 3 cm in diameter on the floor. The number of head dips registered in a 10 min session was recorded by an observer blinded to the experimental treatment. In addition, the locomotor activity of the mice was recorded by an overhead charged-coupled device camera using LimeLight video tracking (ActiMetrics, Wilmette, IL, United States).

### Light-dark test

The light-dark box consisted of a light zone (20 × 20 × 25 cm) and a dark zone (20 × 20 × 25 cm, with black walls and floor), separated by a partition with a single opening (5 × 8 cm) to allow the mice to pass from one zone to the other. Illumination in the light zone was 500 lx, while that in the dark zone was prevented by covering with a lid. A mouse was placed in the center of the light zone at the beginning of the test and the total time it spent in the light zone was recorded for 10 min.

### Cell lines

The human acute myeloid leukemia cell line THP-1 was obtained from the RIKEN BioResource Center (Ibaraki, Japan), and the mouse macrophage cell line RAW264.7, was obtained from the European Collection of Authenticated Cell Cultures (Salisbury, United Kingdom). THP-1 cells were maintained in RPMI1640 medium (Thermo Fisher Scientific, MA, United States) supplemented with 10% fetal bovine serum (FBS) and 10 mg/ml of 1% (v/v) penicillin-streptomycin (Sigma-Aldrich, MO, United States). RAW264.7 cells were maintained in Dulbecco’s modified Eagle’s medium (DMEM) (Thermo Fisher Scientific, MA, United States) supplemented with 10% FBS and 10 mg/ml of 1% (v/v) penicillin-streptomycin.

### *In vitro* experimental schedule

THP-1 cells were seeded at a density of 2 × 10^4^ cells/cm^2^ into 24 well plates. Phorbol 12-myristate 13-acetate (PMA) at a concentration of 200 nM was added to induce differentiation of the cells into macrophages and incubated for 24 h at 37°C in humidified air supplemented with 5% CO_2_. The cells were then exposed to LPS (1.0 µg/ml) and HET (50, 100, and 300 ng/ml) and cultured for another 24 h. The culture supernatant was collected and the concentration of IL-6 secreted in it was measured. Likewise, the RAW264.7 cells were seeded at a density of 5 × 10^3^ cells/cm^2^ into 24 well plates and incubated for 24 h. The cells were exposed to LPS (1.0 µg/ml) and HET (50, 100, and 300 ng/ml) for 24 h. The concentration of IL-6 in the culture supernatant was determined.

### Measurement of IL-6 concentrations in serum and culture medium

IL-6 concentrations in serum and culture medium were determined by enzyme-linked immunosorbent assay (ELISA) using the mouse IL-6 antibody pair or human IL-6 antibody pair (Thermo Fisher Scientific Inc., Cleveland, OH, United States) according to the manufacturer’s instructions. The assay range for human IL-6 was 2–200 pg/ml. The assay range for mouse IL-6 was 4–500 pg/ml.

### Statistics

Values were expressed as mean ± standard error of mean (SEM). The data were analyzed by Student’s t-test or one way and two-way analysis of variance (ANOVA), followed by Dunnett’s test or Tukey-Kramer test (StatView 5.0; Abacus Concepts, Berkely, CA, United States) to determine differences among the groups. *p* < 0.05 was considered as statistically significant.

## Results

### Effect of HET on LPS-induced anxiety-like behaviors in the light-dark test

**FIGURE 1:**
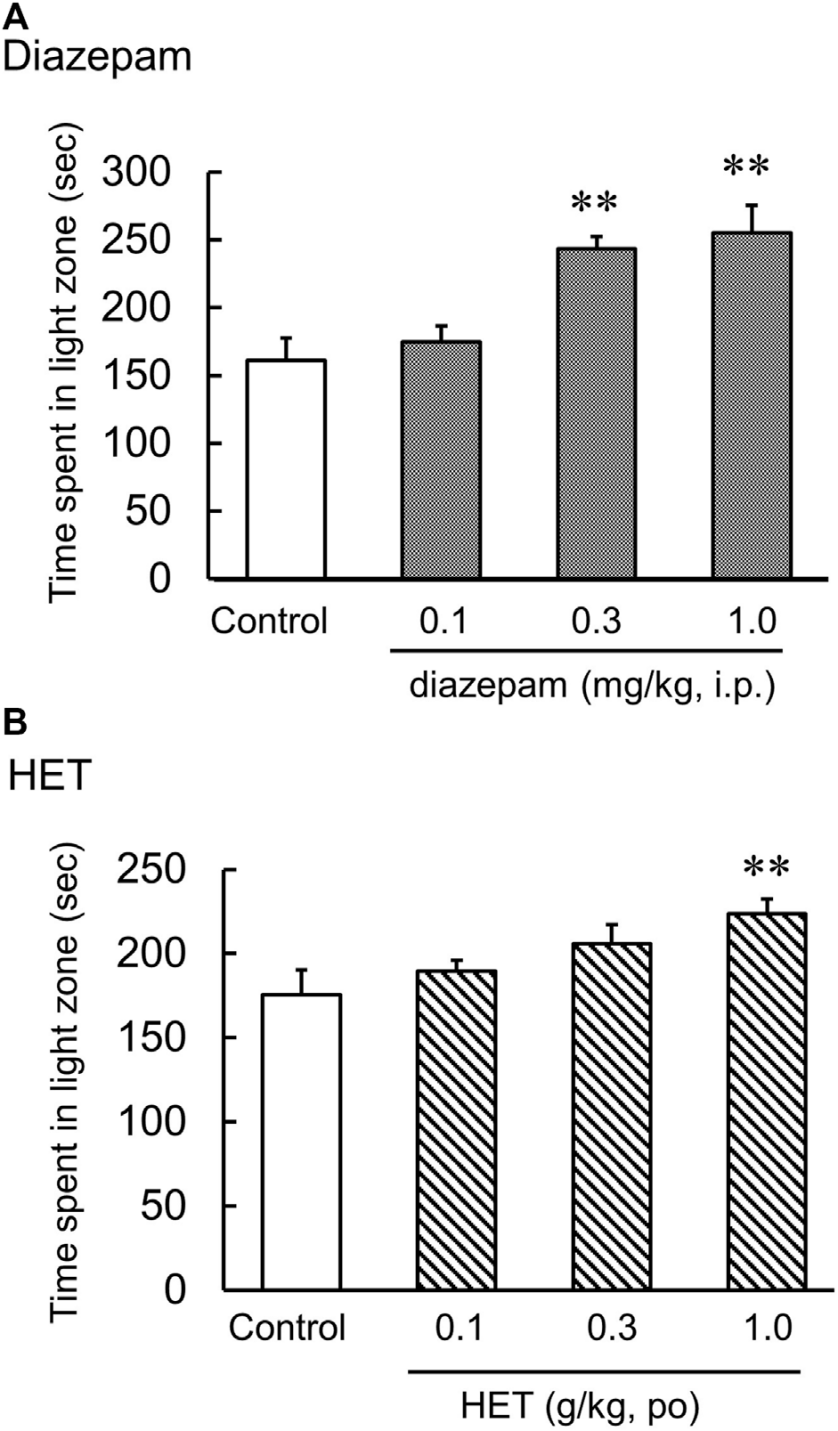
Results of the light-dark test. Effect of diazepam **(A)** and hochuekkito (HET) **(B)** on the time spent in the light zone, that is, showing anxiolytic-like behavior in control mice, mice single administered diazepam and mice administered HET for 2 weeks. Data are shown as mean ± standard error of mean (SEM); *n* = 6–8 per group. **: *p* < 0.01 (vs. control).

In the light-dark test, the light-dark box represents an anxiogenic challenge that tests the conflict between the desire to explore new environments and aversion to brightly lit zone. Diazepam significantly increased the time spent in the light [F (3, 20) = 10.02, *p* < 0.01] (Figure 1A). Additionally, HET also significantly increased the time spent in light [F (3, 26) = 3.81, *p* < 0.05] (Figure 1B). In contrast, LPS-treatment significantly reduced the amount of time the mice spent in light. In LPS-treated mice, HET significantly increased the time spent in light [LPS: F (1, 13) = 2.89, *p* = 0.11; HET: F (1, 13) = 3.49, *p* = 0.08; LPS×HET: F (1, 13) = 2.75, *p* = 0.12] (Figure 3A).

### Effect of HET on LPS-induced anxiety-like behavior in the hole-board test

**FIGURE 2:**
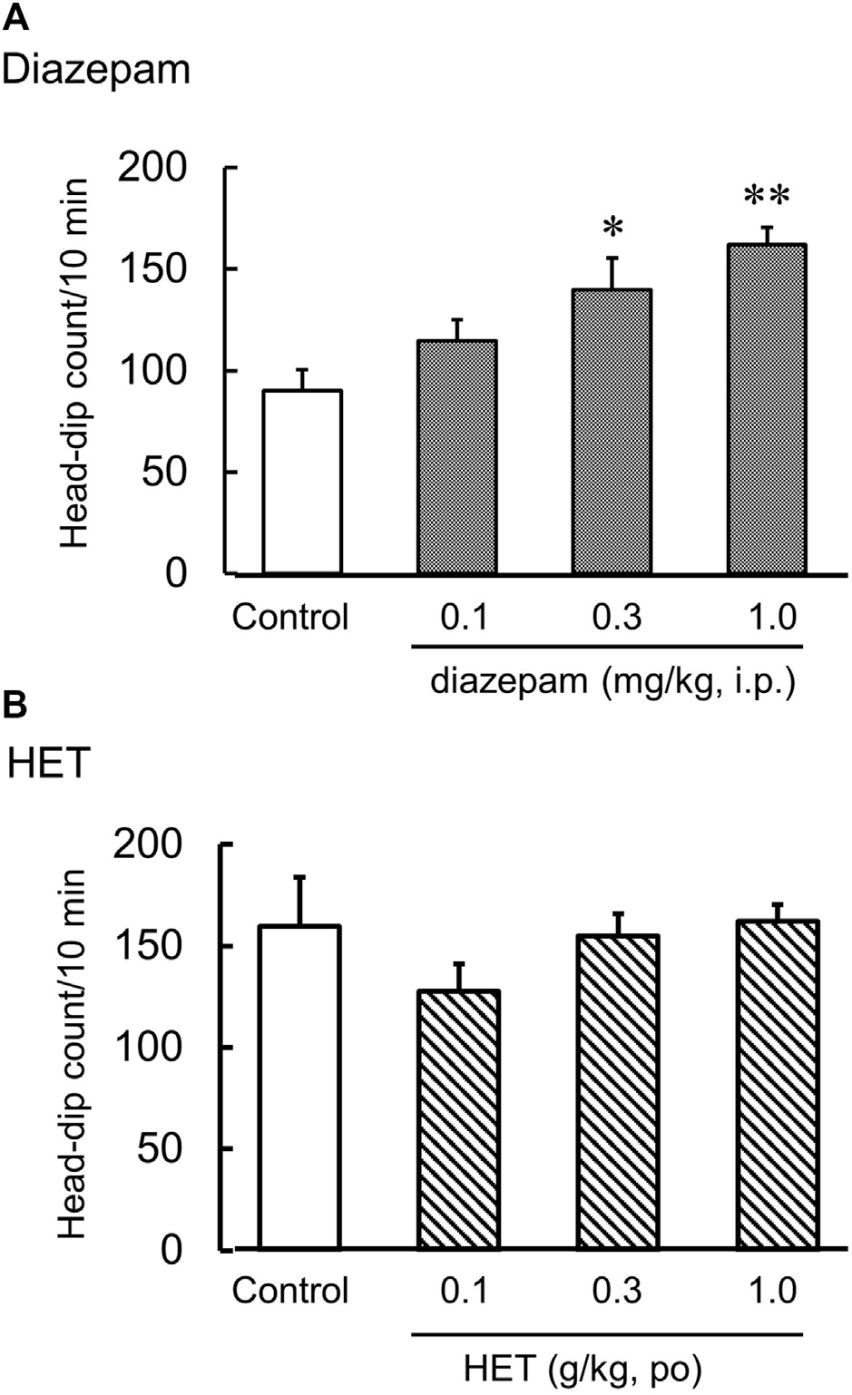
Results of the hole-board test. Effect of diazepam **(A)** and hochuekkito (HET) **(B)** on the number of head dips in control mice, mice single administered diazepam and mice administered HET for 2 weeks. Data are shown as mean ± standard error of mean (SEM); *n* = 6–8 per group. *: *p* < 0.05 (vs. control), **: *p* < 0.01 (vs. control).

**FIGURE 3:**
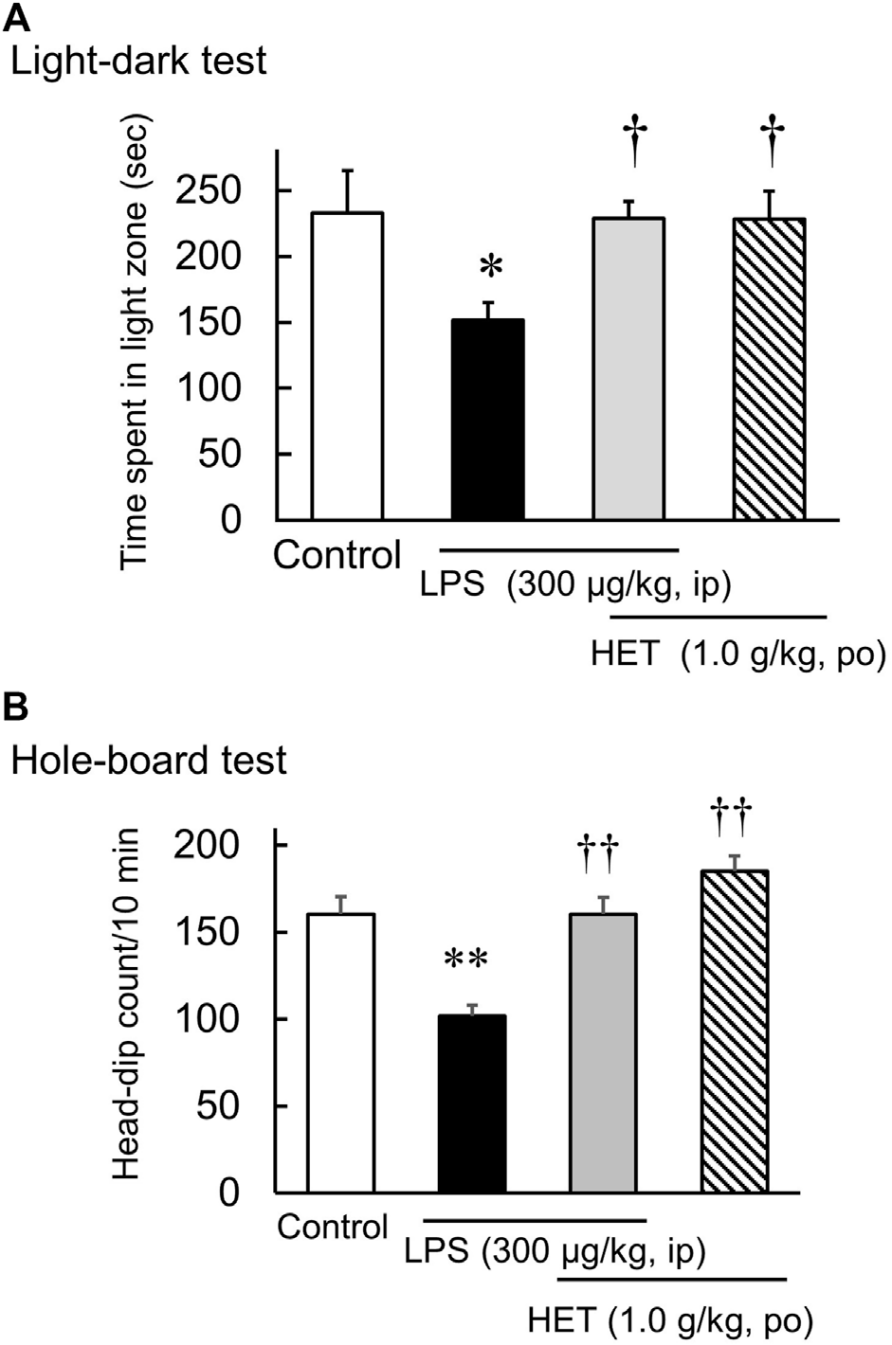
Results of the hole-board test and light-dark test conducted after liposaccharide (LPS)-treatment. Preventive effect of hochuekkito (HET) on the number of head-dips of hole-board test **(A)** and the time spent in the light zone of light-dark test **(B)** in control and LPS-treated mice, and mice before LPS-treatment. Data are shown as mean ± SEM; *n* = 7–8 per group. *: *p* < 0.05 (vs. control), **: *p* < 0.01 (vs. control), ^†^: *p* < 0.05 (vs. LPS), ^††^: *p* < 0.01 (vs. LPS).

The hole-board test is an anxiogenic challenge to assess emotionality and anxiety responses to stress in animals. Diazepam significantly increased the number of head dips in naïve mice [F (3, 20) = 7.33, *p* < 0.01] (Figure 2A). HET did not change the number of head dips in naïve mice [F (3, 22) = 1.06, *p* = 0.39] (Figure 2B). In contrast, LPS-treated mice showed significantly fewer head dips, and HET significantly reversed the number of head dips in LPS-treated mice [LPS: F (1, 15) = 17.67, *p* < 0.01; HET: F (1, 13) = 10.28, *p* < 0.01; LPS×HET: F (1, 13) = 1.07, *p* = 0.32] (Figure 3B). None of the drugs showed a significant effect on locomotor activity in mice at 24 h after LPS treatment (data not shown).

### Effect of glycyrrhizin on LPS-induced anxiety-like behavior in the hole-board test and the light-dark test

**FIGURE 4:**
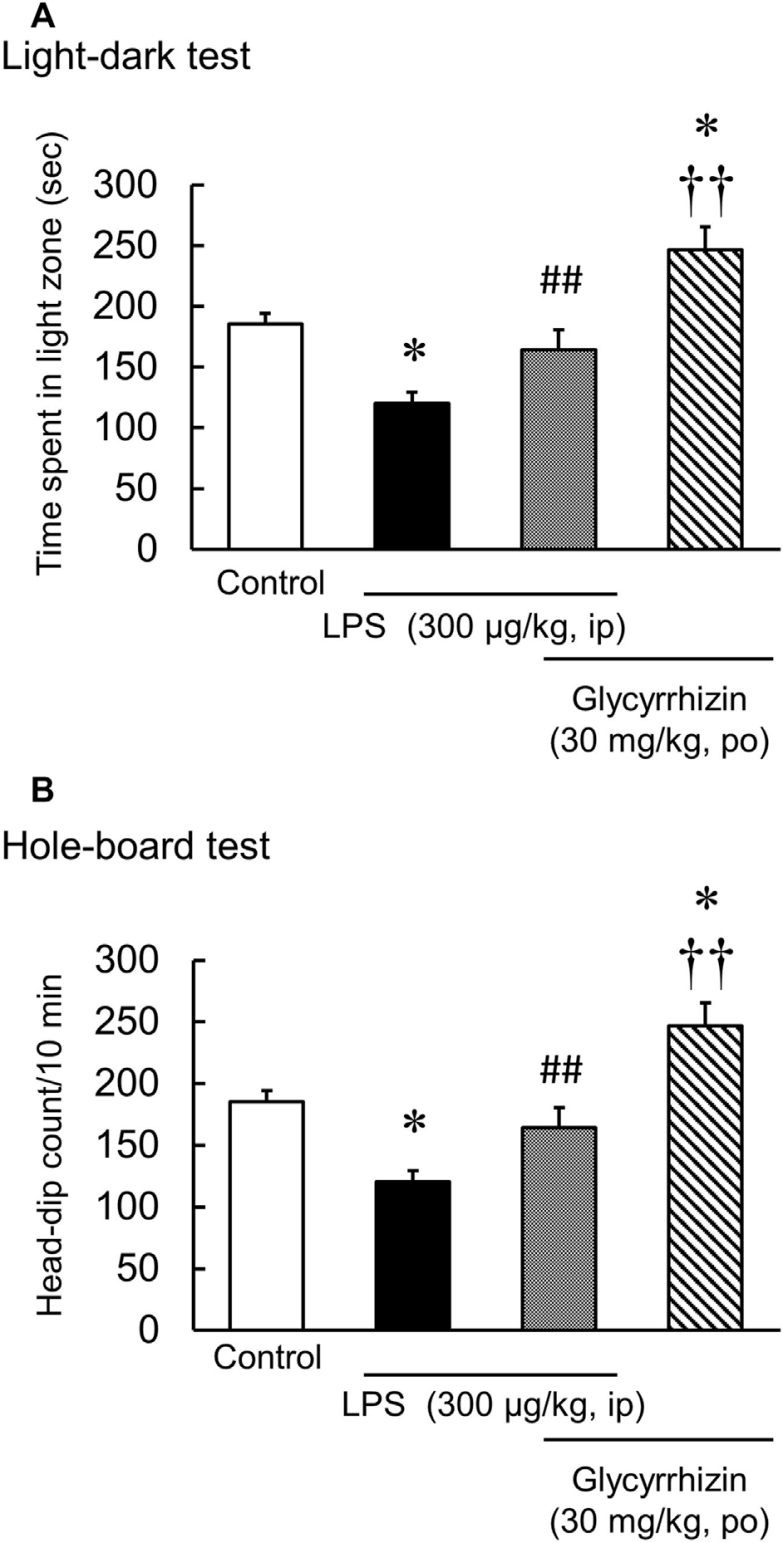
Results of the hole-board test and light-dark test conducted after liposaccharide (LPS)-treatment. Preventive effect of glycyrrhizin on the number of head-dips of hole-board test **(A)** and the time spent in the light zone of light-dark test **(B)** in control and LPS-treated mice, and mice before LPS-treatment. Data are shown as mean ± SEM; *n* = 6 per group. *: *p* < 0.05 (vs. control), ^††^: *p* < 0.01 (vs. LPS), ^##^: *p* < 0.01 (vs. glycyrrhizin).

Glycyrrhizin (30 mg/kg) significantly increased the number of head dips and the time spent in light in naïve mice [Hole-board test: F (3, 20) = 3.89, *p* < 0.05; Light-dark test: F (3, 20) = 2.14, *p* = 0.13] (Table 1). In contrast, glycyrrhizin (30 mg/kg) significantly reversed the number of head dips t and the time spent in the light in LPS-treated mice [Hole board test: LPS: F (1, 11) = 8.21, *p* < 0.05; HET: F (1, 11) = 3.74, *p* = 0.08; LPS×HET: F (1, 11) = 0.11, *p* = 0.75]; Light-dark test: LPS: F (1, 11) = 35.73, *p* < 0.01; HET: F (1, 11) = 10.07, *p* < 0.01; LPS×HET: F (1, 11) = 0.12, *p* = 0.73] (Figure 4).

**TABLE 1:** Results of the light-dark test and the hole-board test. Effect of glycyrrhizin on time spent in the light zone and the number of head dips in control mice, mice administered glycyrrhizin for 2 weeks. Data are shown as mean ± standard error of mean (SEM); *n* = 6 per group. *: *p* < 0.05 (vs. control).

Treatment	Dose (mg/g)	Head-dip counts
(A) Hole-board test		
Control	—	152.0 ± 18.1
Glycyrrhizin	10	186.0 ± 14.7
	30	250.8 ± 32.3*
	50	157.8 ± 22.8

Treatment	Dose (mg/g)	Time spent in light zone (sec)
(B) Light-dark test		
Control	—	170.8 ± 16.8
Glycyrrhizin	10	181.7 ± 23.8
	30	247.0 ± 23.2*
	50	184.5 ± 29.1

### Effect of HET the serum IL-6 levels of LPS-induced mice

**FIGURE 5:**
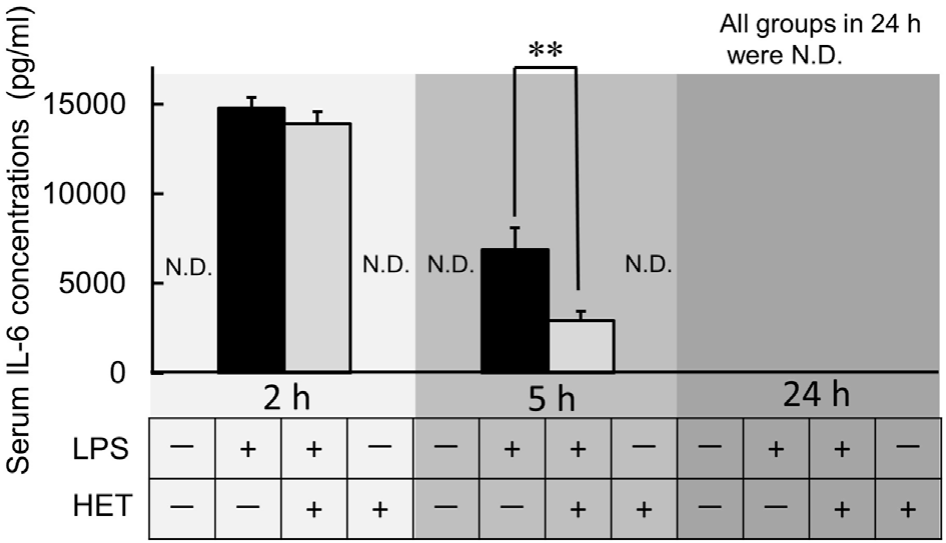
Effect of HET on serum interleukin-6 (IL-6) levels in mice at 2, 5, and 24 h after LPS treatment. ND: not detected. Data are shown as mean ± SEM; *n* = 6–10 per group. **: *p* < 0.01 (vs. LPS).

LPS-treatment caused a significant increase in IL-6 serum concentrations after 2 and 5 h (Figure 5). In all LPS-treated mice, the serum level of IL-6 was below the minimum limit of detection after 24 h. HET did not affect LPS-induced increase in serum IL-6 after 2 h. However, it significantly reduced LPS-induced serum IL-6 increase after 5 h compared to control (*t*-value = 2.964, *p* < 0.01).

### Effect of HET on LPS-induced IL-6 secretion by macrophages

**FIGURE 6:**
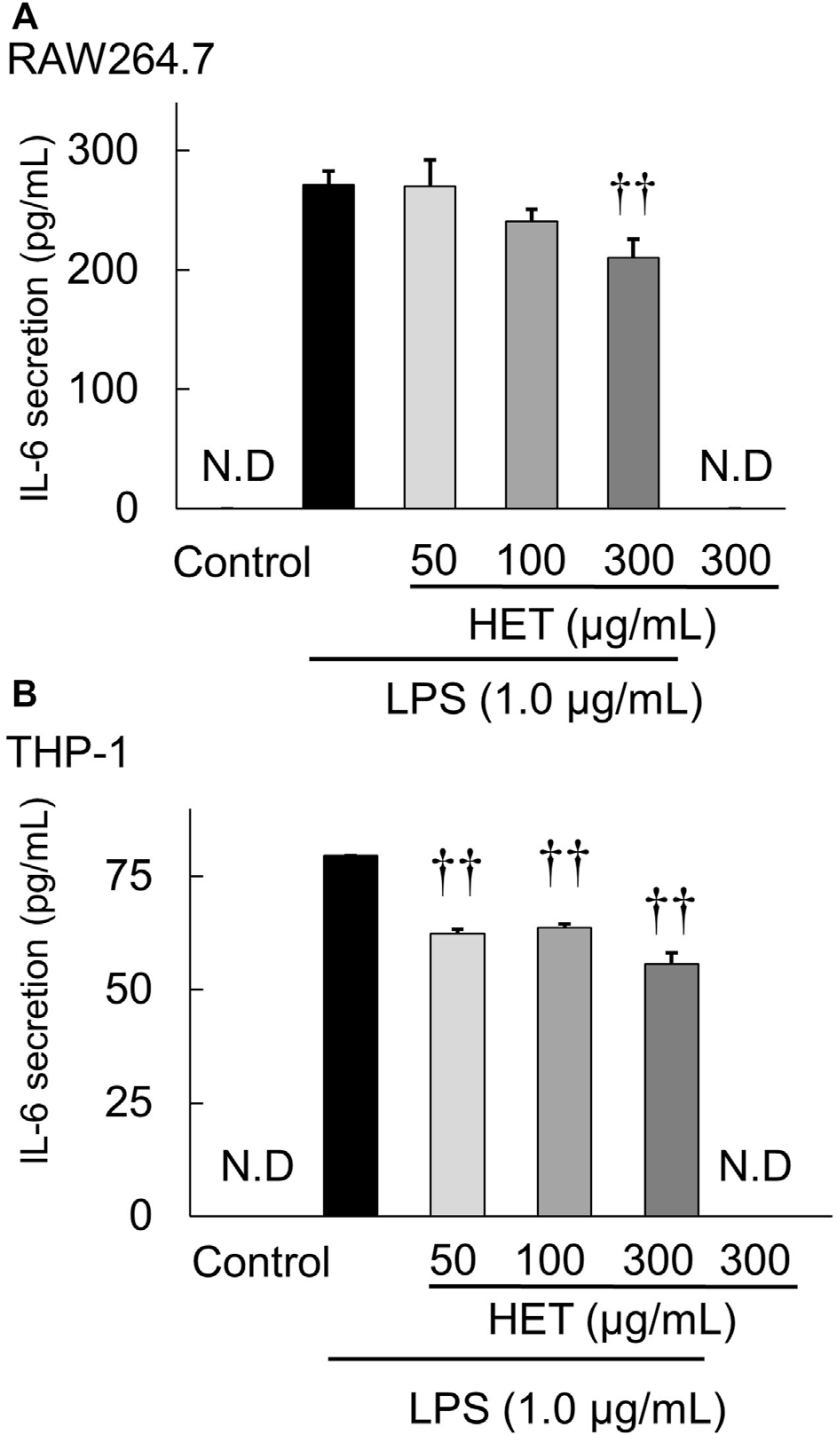
Effect of HET on interleukin-6 (IL-6) secretion from LPS-stimulated RAW264.7 **(A)** and THP-1 **(B)** macrophages incubated in the presence or absence of various concentrations (50–300 µg/ml) of HET. Data are shown as mean ± SEM; *n* = 4 per group. ^††^: *p* < 0.01 (vs. LPS alone).

THP-1 and RAW264.7 cells were used to investigate the effect of HET on LPS-induced IL-6 secretion by macrophages. Exposure of HET at concentrations of 0, 10, 50, 100, and 300 μg/ml did not affect the proliferation of THP-1 cells or RAW264.7 cells (data not shown). LPS exposure at a concentration of 1.0 µg/ml significantly increased IL-6 secretion by THP-1 and RAW264.7 cells. HET significantly suppressed LPS-induced increased IL-6 secretion by RAW264.7 (Figure 6A) [F (3, 12) = 10.325, *p* < 0.05] and THP-1 (Figure 6B) [F (3, 9) = 71.260, *p* < 0.01)] cells.

## Discussion

Several studies have shown that LPS induces anxiety-like behavior in mice (Choubey et al., 2019; Jiang et al., 2019; Sabedra Sousa et al., 2019). An increase in inflammatory markers may induce anxiety-like behavior in animal models (Sulakhiya et al., 2016). We previously reported that LPS treatment increased the production of the pro-inflammatory cytokine IL-6 in the serum, and IL-6 and TNF-α in the hippocampus (Kitamura et al., 2019). In this study, HET was shown to ameliorate anxiety-like behavior (through the light-dark test and hole-board test) and decrease IL-6 levels in LPS-treated mice. Peripheral IL-6 plays an important role in depression-like phenotype after social defeat stress (Yang et al., 2015). Additionally, elevated IL-6 levels in serum are strongly correlated with depression and anxiety symptoms (Hodes et al., 2016). Several reports have shown that blockade of IL-6 signaling (for example, through anti-IL-6 receptor antibody) can be a potential therapeutic strategy for psychiatric disorders (Zhang et al., 2017; Zhou et al., 2017). Therefore, we can assume that IL-6 plays a role in anxiety and depression.

In this study, we used the light-dark test and the hole-board test. These behavioral tests have been previously used to assess anxiogenic and anxiolytic states. A single administration of diazepam, a typical anxiolytic drug, significantly increased time spent in the light compared with the vehicle group. This finding confirmed the anxiolytic-like action of diazepam reported previously (Bourin and Hascoët 2003). According to previous studies, the number of head-dips was also significantly increased by treatment with diazepam in the hole-board test (Takeda et al., 1998). Here, we observed the anxiolytic effect of diazepam with the light-dark test and hole board test. Therefore, these procedures were effective for the evaluation of anxiety-like behaviors in this study.

HET is known to reduce rhinovirus-induced secretion of IL- 6 from human tracheal epithelial cells *in vitro* (Yamaya et al., 2007). Treatment with HET has been shown to decrease serum IL-6 in patients with chronic obstructive pulmonary disease (Tatsumi et al., 2009). Therefore, we can safely assume that HET modulates IL-2 levels. The production of IL-6 in the blood is carried out by immunocompetent cells, such as monocytes and macrophages (Horii et al., 1988). Therefore, we focused on the effects of HET on macrophages. We found that HET decreased LPS-induced IL-6 expression and secretion in human and mouse macrophages, similar to the findings with the mouse models used in this study. These results are consistent with those of a previous study (Yae et al., 2012). Furthermore, HET significantly reduced LPS-induced serum IL-6 levels in mice after 5 h. Therefore, we postulate that HET improves LPS-induced anxiety-like behavior by modulating serum IL-6 levels at an early stage of inflammation.

Regarding the active components responsible for the effects of HET, several major chemical components have been previously identified (Kao et al., 2001; Yamaya et al., 2007). In this study, we especially focused on glycyrrhizin (Lai et al., 2020). We have shown that glycyrrhizin improved LPS-induced anxiety-like behavior. These results suggest that glycyrrhizin could be one of the main constituents of HET that contributes to anxiolytic-like actions under inflammatory conditions. Moreover, HET is composed of ten crude drugs. Among them, ginseng is reported to prevent psychosocial stress-induced psychiatric disorders (Ben-Azu et al., 2020). Ginseng’s main components suppressed immobilization stress and immobilization stress-induced serum levels of IL-6. The anxiolytic effects were shown to occur via the γ-aminobutyrate_A_ (GABA_A_) receptor and serotonergic receptor. Therefore, several crude drugs composed HET may contribute to the anxiolytic-like effects observed in this study.

Toll-like receptors (TLRs) are pattern recognition receptors localized on the cell surface of macrophages and other innate immune cells and play an important role in host defense responses (Fitzgerald and Kagan 2020). LPS, a major component of the extracellular membranes of gram-negative bacteria, is one of the best-studied immunostimulatory exogenous ligands for TLR4. LPS can induce systemic and local tissue inflammation (Lu et al., 2008), and TLRs are an integral part of inflammatory responses. LPS activates macrophages and microglia by selectively stimulating TLR4 and triggering the nuclear factor-kappa B (NF-κB) and mitogen-activated protein kinase pathways (Fu et al., 2013), resulting in the excessive production of pro-inflammatory cytokines (Kim et al., 2012; Wang et al., 2014). It was reported that HET significantly suppressed the increase in LPS-induced TNF-α, a pro-inflammatory cytokine, in human macrophage cell lines, including that used in our study (Isago et al., 2021). HET may also ameliorate inflammation-induced anxiety by inhibiting TLR4 signaling. Moreover, glycyrrhizin is a natural inhibitor of high mobility group box 1 (HMGB1) which has exerted neuroprotective effect against several HMGB1 mediated pathological condition (Mollica et al., 2007). When released into the extracellular settings, HMGB1 exerts biological activity mainly via interaction with its prominent binding partner toll-like receptor-4 (TLR4) and receptor for advanced glycation end products. Thus, it is possible that HET suppress the inflammatory responses included HMGB1/TLR-4/NF-κB signaling pathway. However, as the NF-κB, HMGB1 and MAPK pathways, which are related signals of TLR4, were not examined in this study, further studies are needed to validate this hypothesis.

In conclusion, we demonstrated that HET ameliorated anxiogenic-like behavior under inflammatory conditions induced by LPS using two different behavioral models. Additionally, it was revealed that glycyrrhizin, one of the chemical constituents of HET, suppressed LPS-induced anxiety-like behavior. We also showed that HET decreased IL-6 expression in LPS-induced mice and LPS-exposed macrophages. In conclusion, our study demonstrated that HET inhibits cytokine release, suggesting that the effect of HET on inflammation-induced anxiety-like behavior involves an anti-inflammatory action through IL-6 suppression in the early stages of inflammation.

## Data Availability

The original contributions presented in the study are included in the article/Supplementary Material, further inquiries can be directed to the corresponding author.

## Abbreviations

HET, Hochuekkito; LPS, lipopolysaccharide; IL-6, interleukin-6; TNF-α, tumor necrosis factor-alpha; IFN-γ, interferon-gamma; IL-1β, interleukin-1 beta; JP, Japanese Pharmacopoeia; DMSO, dimethyl sulfoxide; DMEM, Dulbecco’s modified Eagle’s medium; FBS, fetal bovine serum; NF-κB, nuclear factor-kappa B; MAPK, mitogen-activated protein kinase; GABA_A_, γ -aminobutyrate_A_; HMGB-1, high mobility group box 1; N.D, not detected.
