# Supplementary figures and images for: Anxiolytic-like effects of hochuekkito in lipopolysaccharide-treated mice involve interleukin-6 inhibition
Source: Front Pharmacol. 2022 Aug 12;13:890048. doi: 10.3389/fphar.2022.890048 (PMC9411515; doi:10.3389/fphar.2022.890048)

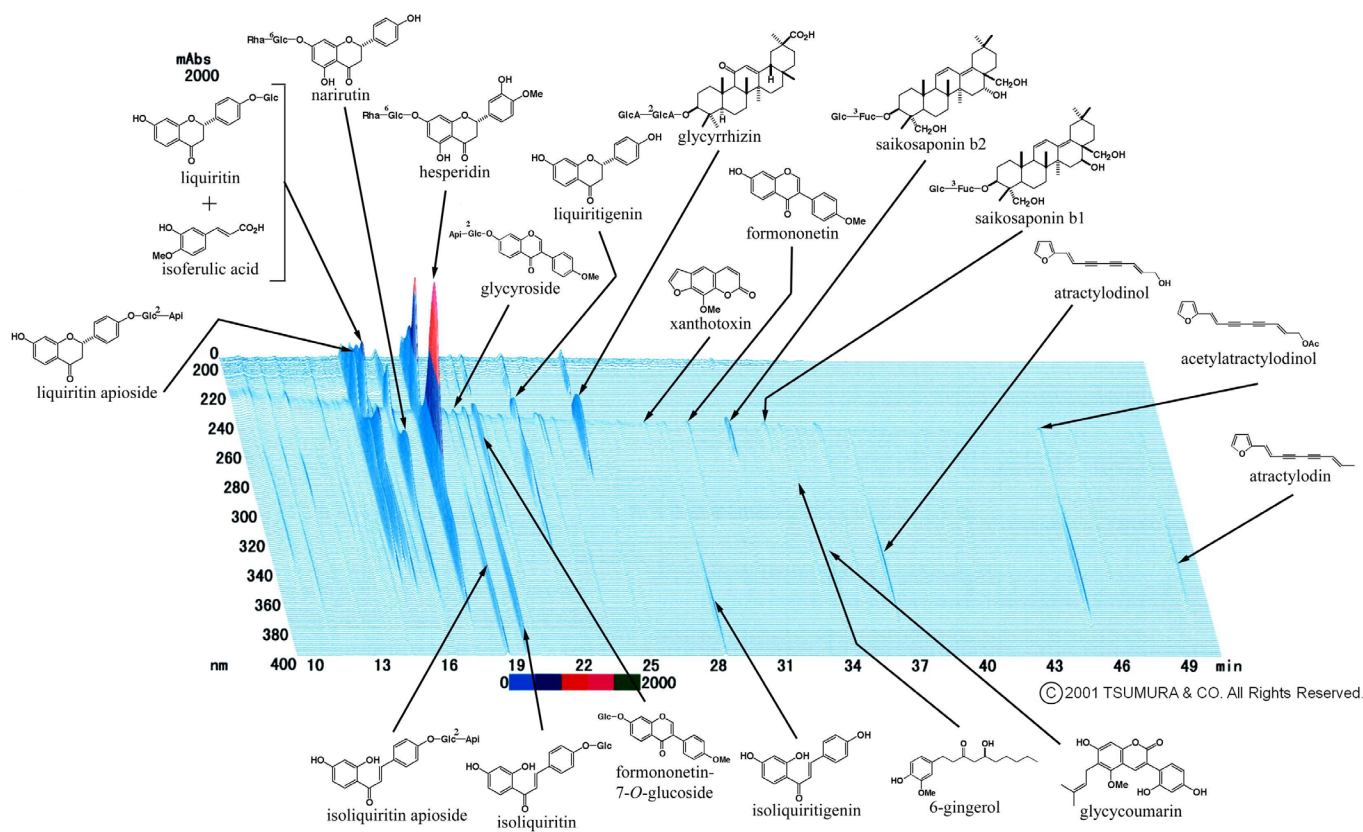

Supplementary Fig. 1. Three-dimensional HPLC profile of hochuekkito

Supplement: Supplementary file 1 [file Image1.pdf]
